# Investigation of thermal exposure in traditional neyshabur bakeries using heat strain and physiological indices

**DOI:** 10.1016/j.mex.2019.02.003

**Published:** 2019-02-12

**Authors:** Somayeh Bolghanabadi, Ali Ganjali, Sahar Ghalehaskar

**Affiliations:** a Department of Occupational Health Engineering, Neyshabur University of Medical Sciences, Neyshabur, Iran; b Students Research Committee, Neyshabur University of Medical Sciences, Neyshabur, Iran; c Instructor of Environmental Health Engineering, School of Health, Jiroft University of Medcial Sciences, Jiroft, Iran

**Keywords:** Heat strain, Bakery, Wet-bulb globe temperature (WBGT), Heat Strain Score Index (HSSI), Physiological index

## Abstract

Bakery is one of the occupations which are exposed to the high shear stress. The incidence of heat discomfort among traditional bakery workers is much more than machinery bakery. The aim of this study was to investigate the rate of heat strain index and temperature of drumhead under physical conditions in this group of workers. This cross-sectional study was conducted on 103 workers of the Bakeries in winter 2018. Heart rate and oral temperature were respectively measured using a heart rate meter and an oral thermometer. The Wet-bulb globe temperature (WBGT) index was recorded and the Heat Strain Score Index (HSSI) was completed simultaneously was used. Data obtained from this study were analyzed by comparing means, *t* test, and Person tests with the SPSS 20 software. The results of the assessment of WBGT, and HSSI showed that 28.69(1.41)and 15.02(2.6) percent of workers exposed to heat stress higher than permissible limits proposed by standard bodies. Also, the present study proved that the mean rate of Heat Strain indicating the presence of strain level 2. According to the results from this study, the oral temperature and drumhead were higher in workers who performed baking activity than other two groups. It is concluded that there is a significant relationship between Heat strain index and Distance of bakery floor ratio to stre.

## Graphical abstract

**Figure:**
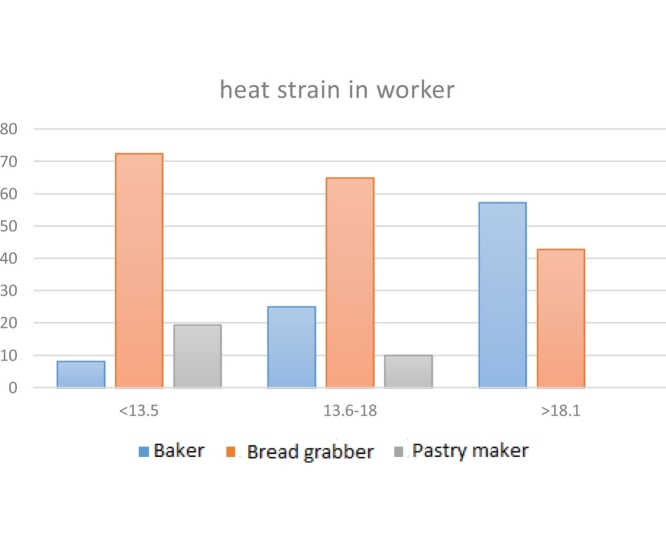

**Specifications Table**

**Table:** 

Subject area	Occoputional Health Engineering
More specific subject area	Heat Strain
Methods name	The applied method in this study is determination to the rate of heat strain index and temperature of drumhead under physical conditions in this group of workers. Heart rate and oral temperature were respectively measured using a heart rate meter and an oral thermometer. The Wet-bulb globe temperature (WBGT) index was recorded and the Heat Strain Score Index (HSSI) was completed simultaneously was used
Name and reference of original method	Dehghan H, Mortzavi SB, Jafari MJ, Maracy MR. Development and validation of a questionnaire for preliminary assessment of heat stress at workplace. Journal of research in health sciences. 2015;15(3):175-81.
Resource availability	The data are available with this article.

## Method details

According to geographical location of Iran (short distance to the equator) has a dry climate [1] and considering that the majority of industrial and non-industrial post heat are faced with heat, and heat from is added to the reason and problems caused by the heat intensifies, and the use of personal protective clothing and equipment according to the type of industry (dust, toxins, microorganisms, ionizing and nonionizing, etc.), their metabolic heat combined with, and the level of body and environment heat exchange limits and ambient temperature and body temperature dropped out, subsequently, deep body temperature exceeds the normal physiological levels and ultimately to prepare for the detection of heat strain [2].

Other aggravating factors for heat-related disorders can be cited such as obesity and overweight, the risk of some chronic diseases, use of certain drugs, failure to comply with heat, low-salt diet, inappropriate work dress [3]. Chronic exposure to hot temperatures caused physiological disorders [4], reduced physical and mental performance and increase in neurological and psychiatric disorders [5], fatigue and dehydration [6] and ultimately reduced productivity [7], an increased incidence risk of accidents [8] and decreased immunity in the work Since harmful effects of working in a hot environment on human health and safety have been demonstrated [9], that if heat stress not controlled, a wide range of symptoms and diseases may be fatal such as mild abnormalities such as burning conditions [10].

Bakery is one of the occupations which is exposed to the high shear stress during summer and inadequate ventilation system [8]. More than 90% of the bakeries in the city of Neyshabur are traditional kind. According to studies on heat strain, the incidence of heat discomfort among traditional bakery workers is much more than machinery bakery. The aim of this study was to investigate the rate of heat strain index and temperature of drumhead under physical conditions in this group of workers.

## Method

This cross-sectional study was conducted on 103 workers of the Bakeries in winter 2018. Samples were selected from people who were without cardiovascular disease, respiratory, infectious diseases, and diabetes and hyperthyroid, people who used cardiovascular drugs, beta blockers, diuretics, antihistamines, were not excluded. Before the study, the consent of all persons to participate in this research was drawn and the workers were assured that the information will remain confidential. The questionnaire containing demographic information including age, height, weight, work experience, education, smoking was completed. The normal body temperature was measured by a mercury thermometer. Heart rate was measured using radial pulse counting. Heat stress in the industry was evaluated for each workstation, for each of the individuals was enrolled in the study. To measure WBGT was used Casella model of digital WBGT. Also the HSSI questionnaire was used to assess the validity and reliability of heat strain that reviewed and approved by Dehghan et al. [11], The questionnaire contains 18 questions where variable temperatures, humidity, air movement, intense sweating, intense thirst, fatigue, sadness, clinical signs, levels temperature, ventilation, type of work wear, work wear color, type of protective equipment, workload, body position, dimensions of work space and location of doing tasks has been studied that score obtained from this questionnaire will be considered as an indicator of heat strain. The first 12 questions were asked of respondents, while the last 5 questions were completed after observation of the workers, scores for each item were multiplied by the effective coefficient of each question. Finally, the scores of the items were summed to yield total score. This index has three levels of risk with numbers less than 13.5 (without strain), numbers between 13.6–18 (containing strain) and numbers over 18.1 (certainly containing strain).

The collected data were analyzed by SPSS software version 24. In the descriptive analysis, the quantitative variables, mean, standard deviation and range were determined. To investigate the relationship between variables, the test of means comparison, including *t* test and chi-square were used.

## Results

The case study sample consisted of 103 workers in Neyshabur - based bakeries. According to the findings, the average age (SD) of the subjects was 37.2 (8.14) with BMI (SD) 24.27 (3.41). Demographic characteristics are presented in Table 1. The mean Wet Bulb Glob Temperature (WBGT) was 28.69 (1.41), which was higher than reported standards. Heat strain indexes, heat stress, and physiological indexes are presented in Table 2. The mean of heat strain obtained was 15.02, indicating the presence of strain level 2. According to the results from this study, the Oral temperature and drumhead were higher in workers who performed baking activity than other two groups.

**Table 1:** Demographic characteristics of in bakery workers.

Variable		Number	Mean	SD	Min	Max
Age	Baker	62	36.5	7.42	26	38
	Bread grabber	20	39.3	9.21	17	39
	Pastry maker	21	37.2	9.24	24	41
	Total	103	37.18	8.14	17	41

BMI	Baker	62	23.81	3.3	16.6	34.2
	Bread grabber	20	25.78	3.02	18.1	28.6
	Pastry maker	21	24.69	3.76	19.4	31.3
	Total	103	24.37	3.41	16.6	34.2

**Table 2:** Mean of physiological parameters and thermal stress indices in bakery workers.

Variable	WBGT	HSSI Index	Oral temperature	Drumhead Temperature	Heartbeat	BMI
Baker	29.4(1.26)	16.07(2.03)	35.99(0.82)	36.4(0.62)	74.62(3.4)	23.81(3.3)
Bread grabber	28.7(1.06)	14.72(1.8)	35.82(0.79)	35.71(0.86)	72.3(4.7)	25.78(3.02)
Pastry maker	26.6(2.21)	12.2(2.75)	35.21(1.19)	35.24(1.13)	73.71(5.35)	24.69(3.76)
Total	28.69(1.41)	15.02(2.6)	35.8(0.94)	36.03(0.77)	73.99(4.1)	24.37(3.41)

According to the results, a significant relationship was found between Wet Bulb Glob Temperature and heat strain index (p < 0.001, r = 0.61). According to Table 3, there was a significant and positive relationship between heat strain and heat stress with all physiological indices except for the mass index which had a reverse and significant relationship with these two indexes.

**Table 3:** Pearson Correlation Coefficient between Physiological Factors and Heat Indicators.

Variable	Drumhead temperature	Oral temperature	Heartbeat	BMI
WBGT	**0.37	*0.26	*0.41	*−0.24
Heat strain (HSSI)	**0.49	*0.56	**0.28	*−0.18

*p < 0.05 and **p < 0.001.

**Chart 1:**
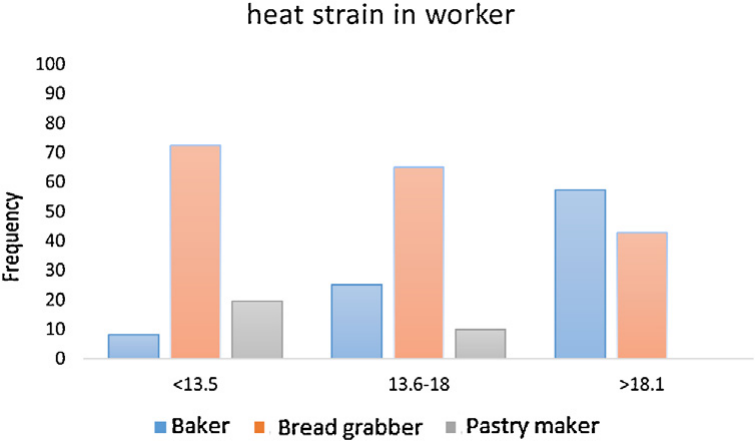
Frequency of heat strain in bakery worker.

In the Chart 1, the heat strain index of bakery workers was determined by the type of task and it was found that most bakers and bread grabbers were definitely in the strain condition.

The heat strain based on the level of the bakery floor relative to the street is presented in Table 4. Based on the results, the highest heat strain was observed in people who work in a building on ground level equal to the street level, and the lowest strain rate in those who had a baker's shop half a meter lower than street floor.

**Table 4:** Heat strain based on the level of the bakery floor relative to the street.

Distance of bakery floor ratio to street	−0.5	Floor	10	20	60	150
Heat strain index	14.15	17.13	14.75	14.79	15.39	15.65

## Discussion

The aim of this study was to determine the amount of thermal exposure by using heat strain index and physiological indices in traditional bakery workers in Neyshabour city. According to the results, more than 80 percent of staff working in the bakery has a WBGT index more than standard value, which indicates the high level of heat stress in these people, and given that the measurements were carried out during the cold season, it has been expected that the amount of heat stress in these people will increase in the summer and cause more heat dissipation. In the study of Hanani et al., Which was carried out in summer, 61.1% of individuals were exposed to heat stress [6], in the study of Jalali et al. [12] that performed in bakeries of Qazvin, the results indicated that High levels of heat stress were found in 63.6% of subjects. Also, the results of this study indicate that the amount of heat strain in all bakery workers (baker, bread grabber, Pastry maker) is an average score of 15.02 in the second level, which has a heat strain, and in the study of Yeganeh et al., the amount of strain was higher than 18 [13], the reason for the difference in this index was season of study, which was reported, in our winter study, the rate of heat strain was lower, but 57% of bakers and 43% of bread grabbers were definitely in heat strain. Also, in our study, the amount of heat strain was higher among the workers with baking and bread grabbing activity than those of the pastry makers, which was due to a longer distance from the heat source and a shorter duration of activity.

Pearson correlation coefficient between heat stress index (WBGT) and heat strain index was 0.61, which indicates a high correlation between these two indicators. Also, the findings indicated that there is a positive and significant relationship between Wet Bulb Glob Temperature with the temperature of drumhead and mouth temperature, which by increasing WBGT levels. Parameter values also increased. In the study of Bolghanabadi [14] and Yeganeh et al. [13], there was a significant relationship between Wet Bulb Glob Temperature and physiological parameters. In the present study, a significant relationship was found between heat strain index and the mouth temperature, which showed that patients with heat strain In terms of mouth temperature, they were not in normal conditions, which was consistent with the study of Bolghanabadi et al. by aiming to investigate the status heat strain and dehydration. In this study, there was a significant and reversed relationship between body mass indexes (BMI). in the study of Bolghanabadi et al. [14], those with heat stress had lower weights compared to the others, and the study by McKein et al. [15] also proved that body temperature of those in Exposure to heat stress was high and weight of these people was lower than those exposed to less heat stress, which is also true in our study. By increasing the body's temperature in workers exposed to heat, the sweating mechanism is activated to overcome this condition and, due to increased thirst, the fluid intake in these people will be more likely to cause digestive diseases in the bakers.

Therefore, most appropriate sanitation measures are needed for these people, which include a suitable ventilation design for this possibility, and a more suitable building design for bakery activity. According to the results from this study, buildings with a height less than 0.5 m have less heat strain and the highest level of strain was on the street floor.

The results of this study and measurements in winter indicate a high heat stress index and heat strain among bakery workers, which is a worrying situation, and further studies should that be carried out based on the type of ventilation used in these occupations and state of the Bakery building with respect to the street level.

## Conflict of interest

There is no conflict of interest.
